# A Call to Improve Usability, Accuracy, and Equity of Self-Testing for COVID-19 and Other Rapid Diagnostic Tests

**DOI:** 10.1089/heq.2023.0020

**Published:** 2023-11-20

**Authors:** Paul K. Drain, Alexandra K. Adams, Larry Kessler, Matthew Thompson

**Affiliations:** ^1^ Department of Global Health and Medicine, University of Washington, Seattle, Washington, USA.; ^2^ Center for American Indian and Rural Health Equity (CAIRHE), Montana State University, Bozeman, Montana, USA.; ^3^ Department of Health Systems and Population Health, University of Washington, Seattle, Washington, USA.; ^4^ Department of Family Medicine, University of Washington, Seattle, Washington, USA.

**Keywords:** COVID-19, rapid diagnostic testing, COVID-19 self-testing, underserved populations

## Abstract

The increasing availability of rapid diagnostic self-tests (RDSTs) for COVID-19 has played an important and increasing role during the pandemic. However, for many underserved communities, RDSTs potential benefits are offset by problems with usability, accuracy, and equity. Given the increased need for and interest in home testing for acute and chronic diseases, including COVID-19, this piece offers ways that regulatory agencies, federal public health agencies, and test developers should engage with diverse communities to ensure equity throughout test development, implementation, and evaluation. Such engagement will ensure maximum personal and public health benefits for current and future RDSTs under real-world conditions.

## Introduction

Diagnostic testing for SARS-CoV-2 has been critical to track COVID-19 cases, monitor the emergence of novel variants, and formulate public health responses. Within 2 years of declaring COVID-19 a global pandemic, hundreds of rapid diagnostic self-tests (RDSTs) for SARS-CoV-2 have been developed.^1^ The increasing availability of RDSTs has implications for policymakers, public health practitioners, and clinical decision makers—including people conducting self-testing. The high demand for use in community- or home-based settings has also altered the regulatory approval procedures. The US Food and Drug Administration (FDA) revised an Emergency Use Authorization (EUA) process,^2^ while the World Health Organization (WHO) issued an emergency use listing procedure for expedited approval.^3^ The US government's adoption and distribution of free RDSTs with relatively quick regulatory approval has benefited many individuals, but created concerns around test usability, accuracy, and equity.

As of May 2022, the US FDA had provided EUA status for at least 18 RDSTs to be used as over-the-counter home-based tests. Before authorizing self-testing for COVID-19, the only FDA-authorized rapid self-test for an infectious disease was an oral HIV swab.^4^ A growing supply of authorized RDSTs for SARS-CoV-2 has fueled an unprecedented interest from the public for access to home-based, self-testing for additional infectious diseases. Our review of the appropriate clinical use and interpretation of COVID-19 RDSTs, revealed significant challenges.^5^

While the evidence of safety and effectiveness may not have been at the usual standards for EUA status, all diagnostic tests were required to meet minimal criteria set by the FDA.^6^ Given the scale and importance of this topic, we review the emerging issues around test (1) usability, (2) accuracy, and (3) equity of access to COVID-19 self-testing (Table 1).

**Table 1.** Recommendations for Improving Food and Drug Administration Emergency Use Authorization Guidelines for Point of Care Tests

	Usability	Accuracy	Equity
FDA guidelines	• Instructions should be less than seventh-grade reading level • May combine with “simple” smart phone app	• *n*=30+ and 30– • >80% positive percent agreement • >98% negative percent agreement • Require 2–3 testing sites	• No guidelines that address equity or access issues
Considerations	• Usability assessment is not required • No field testing, labels, instructions, used, etc.	• Population diversity is not required • Case control designs are known to overestimate test accuracy • No consideration to spectrum of disease (e.g., viral shedding, other clinical, or patient variations) • Minimal consideration of WHO Assured Criteria^a^	• Online test registration for some tests • Internet access or phone access for some tests • Access to mail or shipping to return some tests • Language comprehension • Privacy concerns regarding user information • Cost
Recommendations	• Apply initial usability standards for testing • Increase postmarket surveillance for testing completion	• Require independent or FDA evaluation of accuracy • Require testing with diverse individuals (in terms of disease spectrum, clinical characteristics, or other factors that could impact accuracy) ensuring adequate performance	• Postmarket surveillance with rural and diverse populations • Translation of all materials into Spanish or other languages relevant to the intended use population • Simplify online registration

^a^ WHO assured criteria.

FDA, Food and Drug Administration; WHO, World Health Organization.

### Usability

First, FDA guidelines included recommendations to ensure the usability of RDSTs, including the need for internal controls, easy-to-follow reference instructions with reading comprehension below a seventh-grade level, and “simple” smart phone applications.^6^ While the FDA encouraged usability studies to evaluate workflow and user comprehension, these data were not as stringently assessed for an EUA versus a full 510(k) approval. Further, the rapid EUA process did not consider how products were labeled, designed, and utilized in the field under different operating conditions. Overall, limited assessments of usability factors for self-testing have impaired the utility of RDSTs in home- and community-settings. For example, when test tube labels are too small or made of material that easily smudges, crucial information can be lost, and the sample result may not be processed.

To improve the usability of RDSTs, we recommend more comprehensive initial usability studies and postmarket surveillance. While postmarket surveillance requirements would be removed with the rescinding of the EUA, we argue that FDA should make such data collection a routine part of EUA granting and, note that the EUAs granted in 2020 are still in effect a full 2 years later. Usability studies should document appropriate specimen collection and test usage among people with varying health literacy and across multiple languages. While the FDA has strong premarket requirements, an emphasis on rigorous postmarket surveillance studies could lead to test improvements and accurate reporting of systematic problems in using tests correctly. Implementation studies of COVID-19 self-testing should decipher how and when tests are being used, as well as by whom and for what indication. To ensure continued, safe use of RDSTs, postmarket studies remain particularly important for equity in underserved populations.

### Accuracy

Second, RDSTs need to retain high diagnostic accuracy when untrained laypersons perform self-testing in their home. The FDA requires prospective clinical data from at least 30 positive and 30 negative participants, as determined by reverse transcription-polymerase chain reaction, for separate self-testing and health care worker-testing authorizations.^6^ Since the EUA does not require representation of diverse populations, achieving a minimum positive percent agreement (≥80%) is a low bar with wide confidence intervals. Furthermore, companies submit their own test performance data to the FDA, without an independent external review. Postmarket studies to evaluate test performance have been encouraged, but thus far have not been required.

Finally, WHO has several “ASSURED” criteria to evaluate key performance metrics for RDSTs, many of which have been overlooked in the US regulatory approval process for self-tests.^7^ While at-home RDSTs have become more widely accessible, use has been lower among persons with lower incomes, less education, older adults, and those who self-identified as Black.^8^

Overall, the clinical accuracy of antigen-based RDSTs have been either comparable or inferior to the data reported in most EUA applications.^9^ We want to highlight the need for independent, third-party corroboration of accuracy and performance, or manufacturer collaboration with the FDA's own laboratories to make independent assessments of accuracy. While this may be too cumbersome for an EUA process, it could be considered as part of the 510k approval process.

To improve the accuracy of COVID-19 self-testing, we recommend that RDSTs be subjected to more rigorous and robust testing among diverse cohorts, including underserved populations. Test performance data should be corroborated by independent parties and across several variants of concern. Since there is a relative abundance of available RDSTs in the United States, more attention should be directed toward ensuring adequate performance during implementation of self-testing strategies. RDSTs that cannot achieve the performance criteria through additional studies should have their EUA status rescinded. Additional performance data should be obtained through more rigorous postmarket self-testing studies to detect and mitigate potential sources of error.

Many studies on home-based testing via the NIH RADx-UP program are still in process and may shed light on these topics for underserved populations.^10^ It is important to capture self-test results in the postmarket setting to inform public health interventions. However, with limited research among end users in real-world situations, it remains difficult for test developers, regulators, and officials to efficiently and equitably focus implementation efforts.

### Equity

Third, equity and access to COVID-19 RDSTs has been problematic, particularly in rural, non-English speaking, low health literacy, and/or underserved communities. In our study, underserved rural Latino and Native American communities faced unique complexities and challenges for self-testing.^11^ Barriers included online test registration, accessing broadband communication for smartphone applications or instructional videos, and traveling to sites for test procurement or drop-off. Thus far, few available home-based RDSTs and swabbing kits have instructions in Spanish or other languages. Furthermore, many tests have confusing or complicated instructions that require beyond seventh-grade literacy level. For some, providing detailed user information, as required for test completion, imposed unacceptable privacy concerns.

Additional challenges included feeling overwhelmed with test-kit materials and instructions, as well as uncertainty or challenges with the testing process (50%); transportation challenges to send test kits in for analysis (30%); challenges with timing such as taking time off work to test, time constraints related to when they can test, and waiting for results (19%); and finally, challenges with the actual test kit components provided to complete the test (16%).

We believe that RDSTs should be accessible to and usable by all people, regardless of their income. Therefore, without ensuring that RDSTs can be adequately used by underserved populations, many people may be inadvertently missing appropriate diagnosis and care.

To improve the equity of COVID-19 RDSTs, we recommend broader translation of instructions, less reliance on mobile technologies, and direct delivery of RDSTs to underserved populations. Spanish translation of instructions should be a minimal requirement of RDSTs, while other language translations should be encouraged. Test developers and the FDA should ensure the product information and instructions meet health literacy requirements before product rollout. Use of mobile technologies should remain optional by providing additional support for test interpretation and clinical actions, and any user data will need to be protected to ensure trust. In underserved populations, community members should be engaged in the design, evaluation, and approval processes for self-testing and RDSTs. Postmarket surveillance studies can help monitor adequate language translations and literacy of test materials, test equity and access to underserved communities, and maintenance of trust.

## Discussion

The potential value of self-tests for COVID-19 include immediate access to diagnostic testing and guiding personal decision-making, but the potential benefits are offset by problems with usability, accuracy, and equity, particularly in underserved populations. Not all these issues are unique to COVID-19, but the scale of these issues are unparalleled. Implications of inaccurate or incomplete COVID-19 test results are inappropriate or unsafe mixing within communities, as well as causing widespread anxiety. Given the scientific and financial successes of RDSTs for SARS-CoV-2, several diagnostic companies are planning to launch self-testing products for other infectious diseases, including influenza A and B. Future multiplex RDSTs that combine SARS-CoV-2, influenza A/B, and Respiratory Syncytial Virus (RSV) will be particularly valuable as Influenza cases have increased and appropriate therapeutics are becoming more accessible.

Regulatory agencies, federal public health agencies, and test developers must engage with diverse communities through test development, implementation, and postmarket test evaluation to ensure optimal usability, accuracy, and equity under real-world conditions.

Learning from the challenges and missteps of COVID-19 self-testing, we have an opportunity to leverage the significant global implementation of self-testing to improve equitable health care delivery.

## Abbreviations Used

EUA: Emergency Use Authorization
FDA: Food and Drug Administration
RDSTs: Rapid Diagnostic Self-Tests
WHO: World Health Organization
